# Personalized disease recurrence modeling using iPSC-derived podocytes in patients with idiopathic nephrotic syndrome

**DOI:** 10.1093/ndt/gfaf045

**Published:** 2025-02-28

**Authors:** Bartholomeus T van den Berge, Martijn van den Broek, Gianluca Di Giovanni, Hanna Debiec, Sharon Gloudemans, Quinty Leusink, Dirk den Braanker, Jack F M Wetzels, Pierre Ronco, Bart Smeets, Jitske Jansen, Rutger J Maas

**Affiliations:** Department of Nephrology, Radboud Institute for Molecular Life Sciences, Radboudumc, Nijmegen, The Netherlands; Department of Pathology, Radboud Institute for Molecular Life Sciences, Radboudumc, Nijmegen, The Netherlands; Department of Pathology, Radboud Institute for Molecular Life Sciences, Radboudumc, Nijmegen, The Netherlands; Department of Pediatric Nephrology, Radboud Institute for Molecular Life Science, Radboudumc, Nijmegen, The Netherlands; Department of Nephrology, Radboud Institute for Molecular Life Sciences, Radboudumc, Nijmegen, The Netherlands; Sorbonne Université and Institut National de la Santé et de la Recherche Médicale, Unité Mixte de Recherche, Paris, France; Department of Pathology, Radboud Institute for Molecular Life Sciences, Radboudumc, Nijmegen, The Netherlands; Department of Nephrology, Radboud Institute for Molecular Life Sciences, Radboudumc, Nijmegen, The Netherlands; Department of Nephrology, Radboud Institute for Molecular Life Sciences, Radboudumc, Nijmegen, The Netherlands; Department of Nephrology, Radboud Institute for Molecular Life Sciences, Radboudumc, Nijmegen, The Netherlands; Sorbonne Université and Institut National de la Santé et de la Recherche Médicale, Unité Mixte de Recherche, Paris, France; Department of Pathology, Radboud Institute for Molecular Life Sciences, Radboudumc, Nijmegen, The Netherlands; Department for Renal and Hypertensive Diseases, Rheumatological and Immunological Diseases, Uniklinik RWTH Aachen, Aachen, Germany; Department of Nephrology, Radboud Institute for Molecular Life Sciences, Radboudumc, Nijmegen, The Netherlands

**Keywords:** circulating permeability factor, podocytes, proteinuria, recurrent focal segmental glomerulosclerosis (FSGS), transplantation

## Abstract

**Background:**

Primary focal segmental glomerulosclerosis (FSGS) is characterized by podocyte injury and treatment-resistant nephrotic syndrome. Recurrence of the original disease after kidney transplantation (rFSGS) occurs in 10%–50% of patients. Unidentified circulating permeability factors (CPF) are likely involved in FSGS pathogenesis. We hypothesized that donor podocyte susceptibility to CPF is also relevant. We developed a personalized model for (r)FSGS using induced pluripotent stem cell (iPSC)-derived podocytes from patients and kidney donors.

**Methods:**

Five patients and their respective living kidney donors were included. Three patients had developed rFSGS, and two patients manifested no symptoms of rFSGS. One patient (P5) had heterozygous mutations in *NPHS2*. Peripheral blood mononuclear cells were reprogrammed to iPSC, and differentiated to podocytes. iPSC-derived podocytes from either patients or donors were exposed to presumed CPF-containing plasma/serum of corresponding patients. Three assays to detect podocyte injury were performed: (i) reactive oxygen species formation, (ii) cellular granularity induction, and (iii) quantitative assessment of F-actin redistribution (FAR), a new quantitative method. Crossmatch experiments with donor iPSC-derived podocytes and patients samples assessed individual susceptibility to CPF-induced injury.

**Results:**

Successful podocyte differentiation was confirmed by morphology and protein expression. Only FAR differentiated consistently between patient and healthy donor samples. All pre-transplant patient samples except P5 caused significant FAR in corresponding patient podocytes. Significant FAR was observed in donor podocytes exposed to corresponding patient samples in the setting of rFSGS, and not in donor podocytes exposed to samples of patients who did not develop rFSGS. Effects of FSGS patient samples on non-corresponding donor podocytes were variable.

**Conclusions:**

*In vitro* assays using iPSC-derived donor podocytes may allow individualized assessment of rFSGS. Prospective studies in a larger cohort are required to validate our findings.

KEY LEARNING POINTS **What was known:**

- Patients with primary focal segmental glomerulosclerosis (FSGS) are at risk of recurrence of the original disease after transplantation (rFSGS), proposedly caused by unidentified circulating permeability factors.
- We hypothesized that donor characteristics may be relevant in rFSGS pathogenesis.

**This study adds:**

- We developed an individualized assay to model rFSGS using kidney donors’ induced pluripotent stem cell–derived podocytes.
- The model showed results consistent with clinical observations in patients with rFSGS.
- Podocytes from different donors manifested variable podocyte injury in response to patient plasma.

**Potential impact:**

- Donor characteristics may be relevant in rFSGS pathogenesis.
- Our proposed assay may assist in development of future strategies to prevent rFSGS.

## INTRODUCTION

Primary focal segmental glomerulosclerosis (FSGS) is a major cause of treatment-resistant nephrotic syndrome [1]. FSGS is considered a primary podocytopathy causing podocyte depletion and characteristic focal and segmental glomerular lesions. The disease course of FSGS is characterized by incomplete proteinuria response to immunosuppressive treatment and proteinuria relapses, and ultimately end-stage renal disease [2, 3]. Progressive proteinuria with onset early after transplantation indicates recurrence of the original disease (rFSGS). Despite intense immunosuppressive drug treatment and plasmapheresis, rFSGS is associated with premature graft failure [4, 5]. The risk of rFSGS has been investigated in several studies, and varied between 10% and 50% [6]. Several clinical factors have been associated with higher rFSGS risk, including initial proteinuria responsiveness to corticosteroids, low serum albumin at presentation and native kidney nephrectomies [7–10]. Prior history of rFSGS has been considered the strongest risk factor, although rFSGS rate was still below 75% in these patients [11]. It is presumed that podocyte injury in (r)FSGS is caused by circulating permeability factors (CPF). Several CPF candidates have been proposed, but so far none have been validated [12, 13]. In addition, *in vitro* assays have indirectly demonstrated CPF activity in FSGS patient plasma, based on effects of plasma on cultured podocytes. However, the responses were heterogeneous and/or assays have not been validated for clinical use [14–17]. There remains an unmet need to accurately predict rFSGS in an individual patient, with most research focusing on identifying CPFs. We hypothesized that pathogenesis of FSGS depends on both presence of the CPF and susceptibility of podocytes. To investigate this hypothesis, we developed a personalized assay to model primary FSGS and rFSGS using induced pluripotent stem cell (iPSC)-derived podocytes (based on [18, 19]) from patients and their living kidney donors. Respective iPSC-derived podocytes were exposed to patient plasma obtained before and after kidney transplantation. Podocyte injury assays were performed to evaluate if *in vitro* effects were consistent with the clinical outcome.

## MATERIALS AND METHODS

### Clinical characteristics of study participants

We included five patients with FSGS and their respective living kidney donors. Detailed characteristics are shown in Table 1. Patients and donors received a code based on order of inclusion. All patients had treatment-resistant nephrotic syndrome in their native kidneys, with FSGS confirmed on kidney biopsy. Genetic testing was performed using next-generation exome sequencing in patients P2, P3, P4 and P5. A genetic cause was identified after transplantation in patient P5, based on compound heterozygous mutations in the *NPHS2* gene. This patient was considered a disease control. No genetic cause was identified in P2, P3 and P4. Patient P3 received a pre-emptive transplantation, before dialysis was mandatory. All other patients were treated with hemodialysis before transplantation. Patient P1 underwent bilateral nephrectomies before transplantation because of refractory nephrotic syndrome. After transplantation, three patients developed rFSGS (P1, P2 and P4), whereas two patients did not have a disease recurrence (P3 and P5). All patients with rFSGS had early onset of nephrotic proteinuria, and graft biopsy showing glomerular abnormalities on light microscopy and podocyte foot process effacement in electron microscopy. Blood plasma and/or serum had been obtained before and after transplantation and stored in the Radboud Biobank (CMO no. 09-073). A positive control sample from a previous study (Rec1B) [20] was used to evaluate the assays in iPSC-derived podocytes.

**Table 1:** Clinical characteristics of patients and donors used for iPSC generation and characteristics of blood samples used for exposures.

Patient or donor code	Sex	Age at Tx (years)	FSGS recurrence	Onset of recurrence (days after Tx)	Sample code	Sample type	Time after Tx	CR or PR	Age at sampling (years)	PP session number	Screat (µmol/L)	eGFR (MDRD/CKD-EPI) (mL/min/1.73 m^2^)	Salb (g/L)	Proteinuria (g/10 mmol creatinine)	Immunosuppressive drugs at time of sampling
Patients															
P1	F	39	Yes	D1	P1A	PF	−1 day		39	1^a^	dd	5/5	30	NA	Tac
					P1B	PF	1 day		39	2	146	35/40	36	8.6	Pred/MMF/Tac
P2	M	49	Yes	D2	P2A	P	−1 day		49		dd	5/5	37	NA	
					P2B	P	3 days		49	1	171	37/40	28	4.0	Pred/MMF/Tac
					P2C	P	13 days		49	7	212	29/31	31	2.8	Pred/MMF/Tac
					P2D	P	119 days	PR	49		154	42/45	35	2.4	Pred/Tac/Aza/RTX (22/8 + 16/10)
P3	F	25	No	NA	P3A	S	−1.5 years		23		285	18/20	41	NA^c^	
					P3B	P	172 days		25		143	39/44	39	0.1	Pred/Tac
P4	M	24	Yes	D1	P4A	P	−1 day		24		dd	6/6	28	NA	
					P4B	PF	3 days		24	1	200	36/39	18	3.4	Pred/MMF/Tac
					P4C	PF	13 days	PR	24	9	185	39/43	27	1.3	Pred/MMF/Tac
P5^b^	M	27	No	NA	P5A	P	−3.0 years		23		273	25/28	24	6.9	
					P5B	P	2.3 years		29		122	61/71	26	0.2	Pred/Tac
Donors															
D1	F	63	NA	NA	D1A	P	9.6 years	NA	72	NA	86	56/62	NA	NA	NA
D2	F	52	NA	NA	D2A	P	125 days	NA	52	NA	84	62/72	NA	NA	NA
D3	M	61	NA	NA	D3A	P	172 days	NA	61	NA	162	38/41	NA	NA	NA
D4	F	56	NA	NA	D4A	P	1.4 years	NA	58	NA	86	59/67	NA	NA	NA
D5	F	57	NA	NA	D5A	P	2.3 years	NA	60	NA	94	53/60	NA	NA	NA
Positive control^d^															
Rec1	M	74	Yes	D3	Rec1B	PF	5 days		74	1	169	35/36	28	4.0	Pred/MMF/Tac

a P1 received one plasmapheresis session prior to transplantation.

b Patient P5 was diagnosed with two heterozygous compound (suspected) pathogenic *NPHS2* variants and serves as a disease control.

c P3 had subnephrotic proteinuria (∼2.0 g/10 mmol) in the months prior to sampling.

d Positive control from den Braanker *et al.* (2020) [20].

CKD-EPI, Chronic Kidney Disease Epidemiology Collaboration; Tx, transplantation; F, female; M, male; P, plasma; S, serum; PF, plasmapheresis material; CR, complete remission; PR, partial remission; PP, plasmapheresis; dd, dialysis dependent; Screat, serum creatinine; Salb, serum albumin; NA, not applicable/no data available; Pred, prednisolone; MMF, mycophenolate mofetil; Tac, tacrolimus; Aza, azathioprine; RTX, rituximab.

Peripheral blood mononuclear cells obtained from patients and kidney donors were used to generate iPSCs. The study was conducted in accordance with the Helsinki Declaration as revised in 2013. Permission for the creation and subsequent use of iPSCs and the acquisition of serum and plasma, and use of stored samples was provided by the local ethical commission for human medical research of the Radboudumc, Nijmegen, The Netherlands (CMO no. NL69759.091.19). Written informed consent from all participants was obtained.

### Cell culture

#### iPSC generation and maintenance

Patient and kidney donor iPSCs were generated and maintained as previously described by Jansen *et al*. [21]. For extended methods, see Supplementary materials and methods.

#### Conditionally immortalized podocytes

Human conditionally immortalized podocytes (ciPOD) [22] were cultured in DMEM-HAM's F12 (Gibco) using an adapted protocol previously described by Veissi *et al*. [23]. For extended methods, see Supplementary materials and methods.

### IPSC differentiation protocol

A hybrid directed differentiation protocol based on Rauch *et al*. and Musah *et al*. [18, 19] was developed to differentiate iPSCs into podocytes. For extended methods see supplementary materials and methods. Chemicals, peptides and recombinant proteins and their respective catalog numbers can be found in Supplementary materials and methods (Supplementary data, Table S1).

### Experimental treatment

iPSC-derived podocytes or human ciPODs were treated with either 10% (v/v) plasma, serum or plasmapheresis material (based on previously published work from our lab [20] in serum-free podocyte medium for 24 h after which subsequent endpoint measurements were performed. For extended methods, see Supplementary materials and methods.

### Podocyte injury assays

Podocyte injury was evaluated with three assays. In all assays, the cellular abnormalities after exposure to FSGS patient plasma or serum were compared with effects of the healthy kidney donor pool; pooled donor plasma or serum (PDP/PDS, *n* = 5).

*Reactive oxygen species formation*

Reactive oxygen species (ROS) formation in iPSC-derived podocytes was quantified as previously described by Veissi *et al*. [24]. For extended methods, see Supplementary materials and methods.

*Granule formation analysis using flow cytometry*

Cellular granularity of iPSC-derived podocytes was analyzed as previously described by den Braanker *et al*. [20]. For extended methods, see Supplementary materials and methods.

*Fiji quantification of F-actin redistribution*

Fluorescence images obtained from patient material exposure experiments were analyzed using custom-made macros in the free open-software Fiji/ImageJ (Fiji version 1.53t) (Zenodo link: 10.5281/zenodo.12517187). For extended methods, see Supplementary materials and methods.

### Immunofluorescent staining

Immunofluorescent staining on iPSC-derived podocytes was performed as previously described by Jansen *et al*. [21]. For extended methods, see Supplementary materials and methods. Primary and secondary antibodies and their respective working dilutions can be found in Supplementary materials and methods (Supplementary data, Table S2).

### Anti-nephrin antibody level measurements

Anti-nephrin auto-antibodies were determined and quantitated using enzyme-linked immunosorbent assay. For extended methods, see Supplementary materials and methods.

### Statistical analysis

All data are expressed as mean ± standard deviation (SD) of three independent experiments, unless stated otherwise. Statistical analysis was performed using an unpaired *t*-test or, when appropriate, a one-way analysis of variance followed by Dunnett's *post hoc* test unless stated otherwise. All graphs were made using GraphPad Prism software version 10 (GraphPad Software).

## RESULTS

### Directed differentiation of iPSCs into podocytes

**Figure 1:**
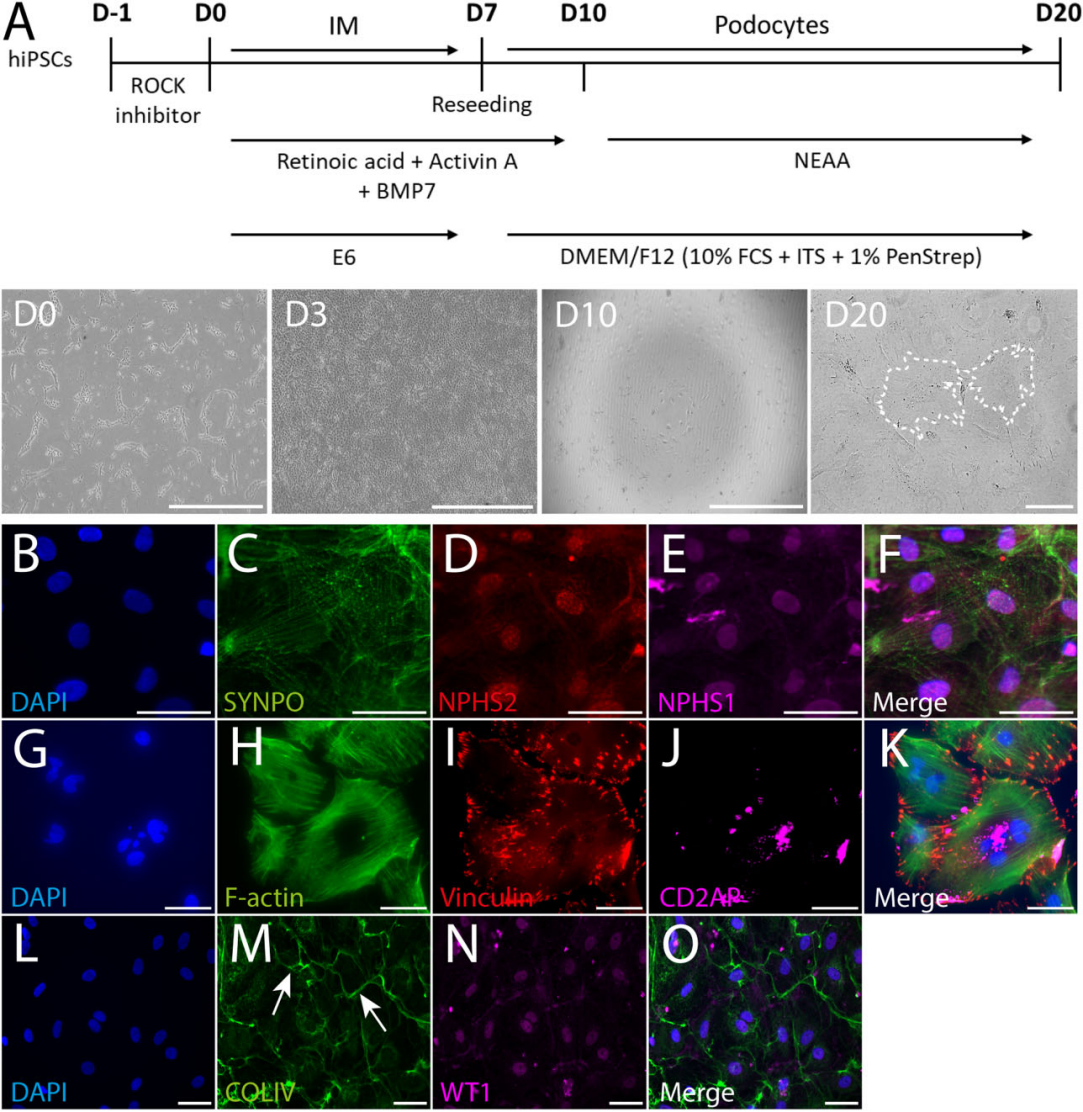
Human iPSC-derived podocytes contain podocyte-specific markers. (**A**) Schematic overview of the iPSC-derived podocyte differentiation protocol. iPSCs were differentiated in 2D into intermediate mesoderm (IM) and reseeded at D7 to stop proliferation and start differentiation into the podocyte lineage. The iPSC-derived cells were cultured for another 13 days to allow for maturation into podocytes. Cell morphology overviews are given at the start of differentiation (D0 and D3), after reseeding (D10) and at the end of the protocol (D20). At D20 two iPSC-derived podocytes are delineated (white dashed lines). BMP7 (bone morphogenetic protein 7), NEAA (non-essential amino acids), FCS (fetal calf serum), ITS (insulin, transferrin, selenium). Scale bars represent 1000 µm (D0, D3 and D10) and 200 µm (D20), respectively. (**B**–**O**) Podocyte-specific marker expression in iPSC-derived podocytes at D20. iPSC-derived podocyte marker expression included synaptopodin (SYNPO) (C), podocin (NPHS2) (D), nephrin (NPHS1) (E), CD2-associated protein (CD2AP) (J) and Wilms’ Tumor 1 (WT1) (N). Vinculin and F-actin staining showed homogenously distributed F-actin fibers ending in vinculin^+^ focal adhesions (H, I). Furthermore, iPSC-derived podocytes deposited COLIV protein pericellularly (white arrows) (M), a main component of the glomerular basement membrane. Scale bars represent 50 µm. Brightness and contrast were adapted on whole images for (B–O).

A schematic overview of the differentiation protocol and cellular morphology during differentiation is given in Fig. 1A. Using this directed protocol, iPSC-derived podocytes at Day 20 (D20) showed expression of synaptopodin (SYNPO) (located along the actin fibers) (Fig. 1C), CD2-associated protein (CD2AP) (Fig. 1J) and Wilms’ Tumor 1 (WT1) (Fig. 1N), as well as the presence of homogenously distributed F-actin fibers ending in vinculin^+^ focal adhesions (Fig. 1H and I). Podocin (NPHS2) and nephrin (NPHS1) showed mostly (peri-) nuclear and slight cytoplasmic stainings (Fig. 1D and E), a pattern similar to that in previous reports in iPSC-derived podocytes [18, 19]. Production of glomerular basement component collagen type 4 (COLIV) could also be observed in our iPSC-derived podocytes (Fig. 1M white arrows, depositions of COLIV were observed pericellular). iPSC-derived podocytes are relatively frequently multinucleated, which is in line with *in vivo* observations (Fig. 1G and L) [25]. IPSC-derived podocyte cell lines from all participants showed consistent differentiation for podocyte-specific markers (Supplementary data, Fig. S1).

### Evaluation of assays to detect CPF-induced podocyte injury *in vitro*

**Figure 2:**
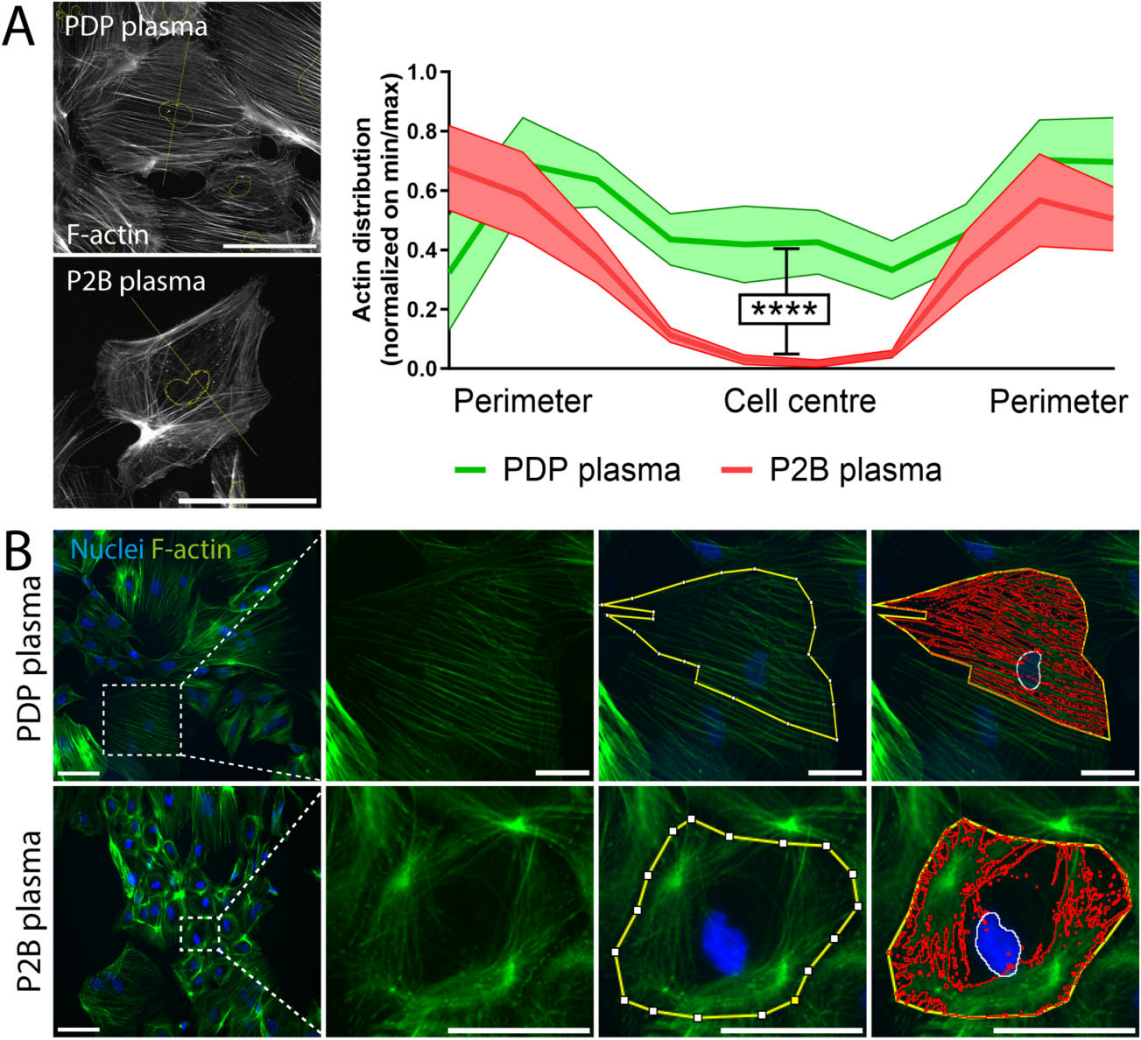
Human iPSC-derived podocytes cytoskeletal rearrangement upon exposure to FSGS recurrence plasma. (**A**) Actin rearrangement was quantified according to the method of Zonderland *et al.* [26]. A statistically significant difference was observed in donor iPSC-derived podocytes (D2) when exposed to either healthy PDP or the donor's respective FSGS recurrence patient plasma sample P2B (*N* = 3). (**B**) Top panel: representative overview of iPSC-derived podocytes exposed to healthy pooled donor plasma (PDP) showing homogenously distributed F-actin fibers. Lower panel: iPSC-derived podocytes exposed to FSGS patient plasma (P2B) showing aberrant F-actin fiber distribution. The third column shows manual singular cell annotation (yellow line). The fourth column shows the processed cell with manual singular cell annotation (yellow line), nuclear selection based on DAPI (white line) and thresholded F-actin^+^ area within the annotated cell (red lines). PDP, pooled donor plasma. Scale bars represent 100 µm and 50 µm for overview and cropped images, respectively. ****P ≤ .0001 (Student's *t*-test). Brightness and contrast were adapted on whole images.

We performed three assays to analyze podocyte injury after exposure to FSGS patient samples, based on previous studies performed in our laboratory: [1] intracellular ROS formation, [2] cellular granularity and [3] quantitative assessment of F-actin redistribution (FAR) [20, 24, 26]. For FAR, we initially tested the method of Zonderland *et al*. [26] for use in our iPSC-derived donor podocytes. We could detect actin rearrangements in donor D2 iPSC-derived podocytes when exposed to patient plasma sample P2B (Fig. 2A), showing clear cytoskeletal changes already prior to quantification. We then adapted and validated this method for more high-throughput analyses in our iPSC-derived podocytes (Fig. 2B), as recently reported using ciPODs [23]. Inter-observer variance of the adapted method between two independent examiners was 2.2% (±12.1% SD), independent of effect size (Supplementary data, Fig. S2). For validation purposes, respective assays were performed on iPSC-derived podocytes from kidney donors, using a positive control FSGS recurrence patient sample (Rec1B) (which caused a strong increase in ciPOD granularity; den Braanker *et al*. [20]). After exposure to the positive control sample, respective podocyte injury assays showed abnormalities in all donor iPSC-derived podocytes (Supplementary data, Fig. S3). Notably, anti-nephrin antibody levels were not significantly elevated compared with pooled donor plasma (Supplementary data, Fig. S4).

### rFSGS modeling *in vitro*

**Figure 3:**
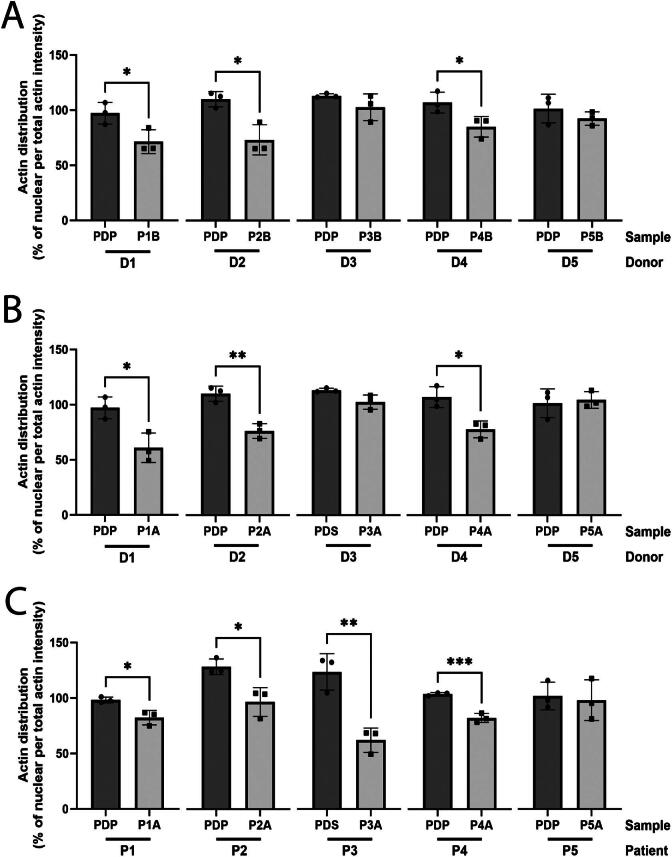
FSGS disease modeling in human iPSC-derived podocytes. (**A**) Donor iPSC-derived podocytes were exposed to their respective post-transplantation (Tx) patient plasmas to mimic the *in vivo* situation in the donor kidney after transplantation. Statistically significant podocyte injury was detected for D1, D2 and D4, but not for D3 and D5 which is in line with the *in vivo* clinical outcomes of these donor kidneys after transplantation (*N* = 3). (**B**) Donor iPSC-derived podocytes were exposed to their respective pre-Tx patient plasmas/seras to evaluate the response of patient podocytes to potential CPF-containing pre-Tx sample. Statistically significant podocyte injury was detected for D1, D2 and D4, but not for D3 and D5, mimicking disease outcome in patients (*N* = 3). (**C**) Patient iPSC-derived podocytes were exposed to their respective pre-Tx patient plasmas/seras to mimic the *in vivo* situation in the native kidney (*N* = 3). PDP, pooled donor plasma; PDS, pooled donor serum. *P ≤ .05; **P ≤ .01; ***P ≤ .001 (Student's *t*-test).

For rFSGS modeling, both ROS formation (Supplementary data, Fig. S5A) and granule formation (SSC) (Supplementary data, Fig. S5B) did not cause significant alterations in iPSC-derived donor podocytes. Significant FAR, however, was detected in donor-derived podocytes (D1, D2, D4) exposed to their corresponding patient plasma obtained at the time of rFSGS onset (*P* ≤ .05), whereas the actin cytoskeleton remained unaltered in donor-derived podocytes (D3 and D5) exposed to their respective non-rFSGS plasma (*P* > .05) (Fig. 3A). The results were thus in correspondence with the observed clinical outcome. We questioned whether podocyte injury observed *in vitro* would follow the disease course as observed in patients. We investigated consecutive samples of patients with rFSGS who reached partial proteinuria remission (P2 and P4). Indeed, plasma samples obtained at the time of reduced proteinuria caused less podocyte injury as assessed by FAR (Supplementary data, Fig. S6). Notably, rFSGS samples did not cause significant podocyte injury in ciPODs, regardless of the assay used (Supplementary data, Fig. S7). We subsequently exposed donor iPSC-derived podocytes to pre-transplant samples of corresponding patient to evaluate potential predictive value of the assay for rFSGS. D1, D2 and D4 showed increased FAR, whereas no change was observed in D3 and D5, which corresponded with the clinical outcome after transplantation (Fig. 3B). Primary FSGS modeling was performed by exposure of patient iPSC-derived podocytes to their respective patient plasma sample obtained before transplantation. The FAR assay detected significant abnormalities in patient iPSC-derived podocytes from P1, P2, P3 and P4, whereas P5 (disease control with genetic FSGS) did not show podocyte injury (Fig. 3C).

### Exposure of iPSC-derived donor podocytes to plasma from other patients: a cross-test for rFSGS?

**Figure 4:**
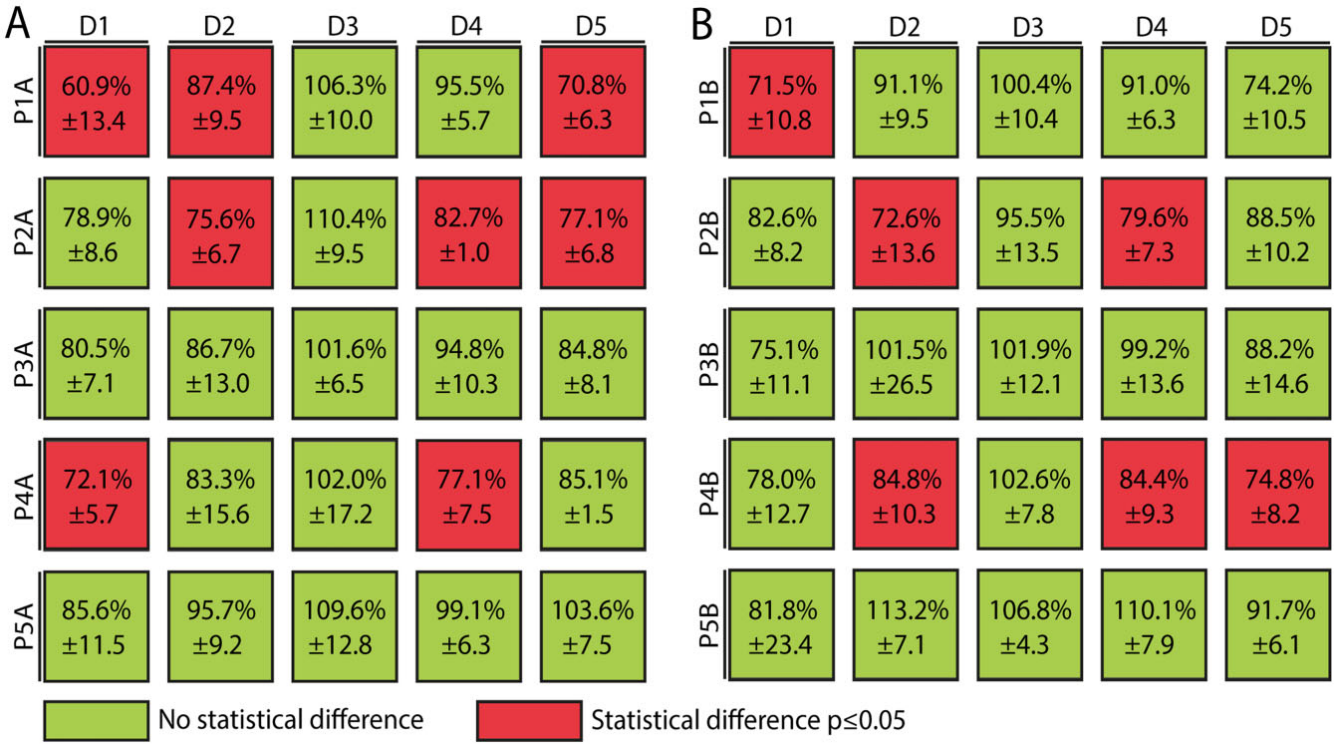
Donor-specific characteristics influence outcome of podocyte injury. (**A**) Donor iPSC-derived podocytes exposed to all pre-transplantation (Tx) patient plasmas. (**B**) Donor iPSC-derived podocytes to all post-Tx patient plasmas. Outcome of the FAR assay is presented as a percentage (nuclear per total cellular actin intensity) where the color code indicates whether there is a statistically significant difference compared with the respective healthy pooled donor plasma (PDP) (*N* = 3) (as statistical tests, Student's *t*-test were performed).

To investigate whether different donor podocytes may indeed manifest different susceptibility to suspected CPF in patient plasma, we performed virtual crossmatch experiments. Respective donor iPSC-derived podocytes were exposed to all patient samples. Figure 4 shows results of the FAR assay using pre-transplant (Fig. 4A) and post-transplant patient samples (Fig. 4B), respectively. Samples from non-rFSGS control P3 (P3A and P3B) did not show any statistically significant differences in combination with any of the donors, nor did the samples from disease control P5 (P5A and P5B). Podocytes from donor D3 did not show statistically significant differences with any of the patient samples, suggesting that the donor may be less susceptible to CPF-induced podocyte injury (Fig. 4A and B). Other donor iPSC-derived podocytes had variable responses to patient samples, suggesting that patient–donor interactions may indeed be relevant.

## DISCUSSION

This study describes a personalized model of podocyte injury in rFSGS, based on effects of patient plasmas on iPSC-derived podocytes from corresponding kidney donors. Although several studies have shown that presumed CPF-containing plasma may cause podocyte injury, this has not been studied at the level of individual patient and kidney donor couples.

We observed that patient plasma obtained at the onset of rFSGS caused significant FAR in podocytes of the corresponding kidney donor, and not in controls who did not develop rFSGS. Similar results were observed using patient plasma obtained before transplantation, suggesting that the assay may be used for pre-transplant prediction of rFSGS. In keeping with clinical observations, all patient-specific podocyte lines manifested significant FAR after exposure to pre-transplant plasma, except in patient P5 who had a confirmed genetic cause of FSGS. Finally, we performed a virtual cross-match for rFSGS, in which respective donor podocyte cell lines were exposed to all patient samples. This experiment confirmed that non-rFSGS plasma did not cause significant podocyte injury in any donor podocyte cell line. Samples of patients who developed rFSGS caused variable podocyte injury in respective donor podocytes. Importantly, podocytes from donor D3 did not manifest podocyte injury in response to any rFSGS sample in the study cohort, and showed limited injury in response to the positive control (Rec1B). These data supported our hypothesis that there is variation between individual kidney donors in podocyte susceptibility to CPF in patient plasma.

rFSGS is considered the prototype of CPF-related podocyte injury. For this reason, many studies have used rFSGS patient plasma to study effects of CPF on cultured podocytes *in vitro* [20, 23, 24]. Although this approach has achieved some positive results, no assays have so far been validated for clinical use. Several explanations have been sought for this lack of success [27]. Samples selected for *in vitro* experiments may not have fully represented the reported clinical phenotype of the patient. We addressed this issue by the inclusion of patients with a clear phenotype of rFSGS and controls with non-rFSGS, inclusion of samples obtained before and after transplantation, and selection of a read-out that resembled clinical observations most closely. Another issue is potential lack of similarity between cultured podocytes and podocytes *in vivo* [28]. In this study, we applied an optimized protocol to culture podocytes from donor iPSC which expressed podocyte-specific markers quite well. Previous studies have successfully used iPSC-derived podocytes for personalized disease modelling, especially in genetic disorders [21, 29]. These data demonstrate that iPSC-derived podocytes maintain genetic characteristics of the original cell line. Admittedly, physiological characteristics of human podocytes are even better represented in kidney organoids, where podocytes interact with other glomerular cells. However, compared with two-dimensional culturing systems, organoid podocytes may be less exposed to CPF in patient plasma, and optimal experimental conditions have not yet been defined [21]. A third issue is a potential lack of sensitivity of *in vitro* models to detect CPF at low concentrations. In this study, we demonstrated podocyte injury using iPSC-derived podocytes using samples that did not cause podocyte injury using ciPODs, suggesting that the iPSC-derived personalized podocyte model may be more sensitive compared with ciPODs.

Several methods have been used to detect podocyte injury after exposure to presumed CPF containing plasma [14–17], but these have not been compared systematically. In this study, we evaluated methods which were previously established in our lab [20, 23, 24]. We did not observe CPF effects on ciPODs nor iPSC-derived podocytes using podocyte granularity and ROS production as read-out. More consistent results were obtained using FAR as read-out in iPSC-derived podocytes. In support of this method, a change in podocyte F-actin cytoskeleton after exposure to CPF containing plasma has been observed previously [14, 15, 30, 31]. Our current report offers a reproducible and quantitative method to quantify FAR, and extends previous results using ciPODs [23]. Notably, we observed variable FAR in respective donor-derived podocyte cell lines after exposure to the same plasma. Remarkably, D3 iPSC-derived podocytes did not manifest significant FAR in response to any patient sample in our study. Podocyte differentiation efficiency was not significantly different between respective iPSC-derived cell lines, and thus cannot account for this observation. In line with our hypothesis, we speculate that reduced donor susceptibility to CPF may explain the absence of FAR. Several relevant genes and pathways relevant for podocyte integrity have been identified, which may be variable between individuals and could serve as an explanation for differences in susceptibility [32, 33].

Recent observations may support our hypothesis. It has been frequently stated that prior rFSGS conveys a very high risk for recurrence in subsequent grafts. Nevertheless, a recent study showed that more than 25% of patients with prior rFSGS did not manifest recurrence in a subsequent graft, irrespective of pre-emptive treatment [11]. In addition, recent studies have identified auto-antibodies targeting nephrin as a potential cause of podocytopathies, including rFSGS [12, 34, 35]. In a recent study, pre-transplant elevated anti-nephrin was detected in a minority (38%) of adult patients with rFSGS, but was highly predictable of rFSGS [36]. In our study, anti-nephrin was not significantly elevated in any of the patient samples (Supplementary data, Fig. S4). Therefore, our results may not pertain to rFSGS associated with anti-nephrin.

Several limitations of our study should be noted. Development of iPS cell lines and directed podocyte differentiation was associated with high cost and is a time-consuming process that may take up to 3 months. Available resources allowed inclusion of five patients and kidney donors. Our study was therefore designed to test a proof of principle, and the results should be confirmed in a larger patient cohort. Compared with non-rFSGS controls, samples of patients with rFSGs were obtained earlier after transplantation. However, serum creatinine levels and immunosuppressive medication were similar. Although genetic testing was not performed in P1, the clinical course with immediate-onset rFSGS and temporary response to plasmapheresis was not compatible with a genetic cause. Furthermore, our observation of improved FAR after therapeutic plasma exchange in patients with rFSGS suggested that the observed changes were indeed disease-specific. Similarly, timing of pre-transplant sampling was different between rFSGS and non-rFSGS patients. Although samples of patients P3 and P5 were not obtained while patients were on dialysis treatment, both had severely reduced eGFR. In addition, patient P5 with genetic FSGS had nephrotic syndrome at the time of pre-transplant sampling. Absence of significant FAR in experiments with this sample suggested that the effects were not caused by the nephrotic state. Our study included different sample types based on availability, including plasma, serum and plasmapheresis fluid. We did not have the opportunity to evaluate effects of different sample types obtained simultaneously. Finally, several discrepancies were observed between pre- and post-transplant samples in the virtual cross-match for rFSGS (Fig. 4). Studies with more patients are needed to evaluate whether pre-transplant samples can indeed be used for rFSGS disease modeling.

Despite these limitations, our data encourage application of the methods in a larger cohort of patients with FSGS and living kidney donors. If the results are confirmed, further studies focusing on identification of potential characteristics associated with podocytes susceptibility to CPF should be performed. In parallel, validation of our results would enable future strategies to prevent rFSGS by assessment of the *in vitro* response to pre-emptive treatment and selection of a kidney donor with reduced podocyte susceptibility to CPF.

In conclusion, this study showed feasibility of personalized rFSGS disease modelling using iPSC-derived podocytes from kidney donors. In addition, our results suggested that donor characteristics may be relevant in rFSGS pathogenesis. This information should be considered in future studies of rFSGS.

## Supplementary Material

gfaf045_Supplemental_Files

## Data Availability

The Fiji macro script used for phalloidin cytoskeleton quantification is available on Zenodo (Zenodo link: 10.5281/zenodo.12517187). The data underlying this article will be shared on reasonable request to the corresponding author.
