# Supplementary material for: Personalized disease recurrence modeling using iPSC-derived podocytes in patients with idiopathic nephrotic syndrome
Source: Nephrol Dial Transplant. 2025 Feb 28;40(9):1736–45. doi: 10.1093/ndt/gfaf045 (PMC12451694; doi:10.1093/ndt/gfaf045)
Supplement: gfaf045_Supplemental_Files [file gfaf045_Supplemental_Files.zip › 875 NDT_Supplemental figures_Personalized disease recurrence modeling using iPSC-derived podocytes in patients with idiopathic nephrotic syndrome_final_cleanversion.docx]

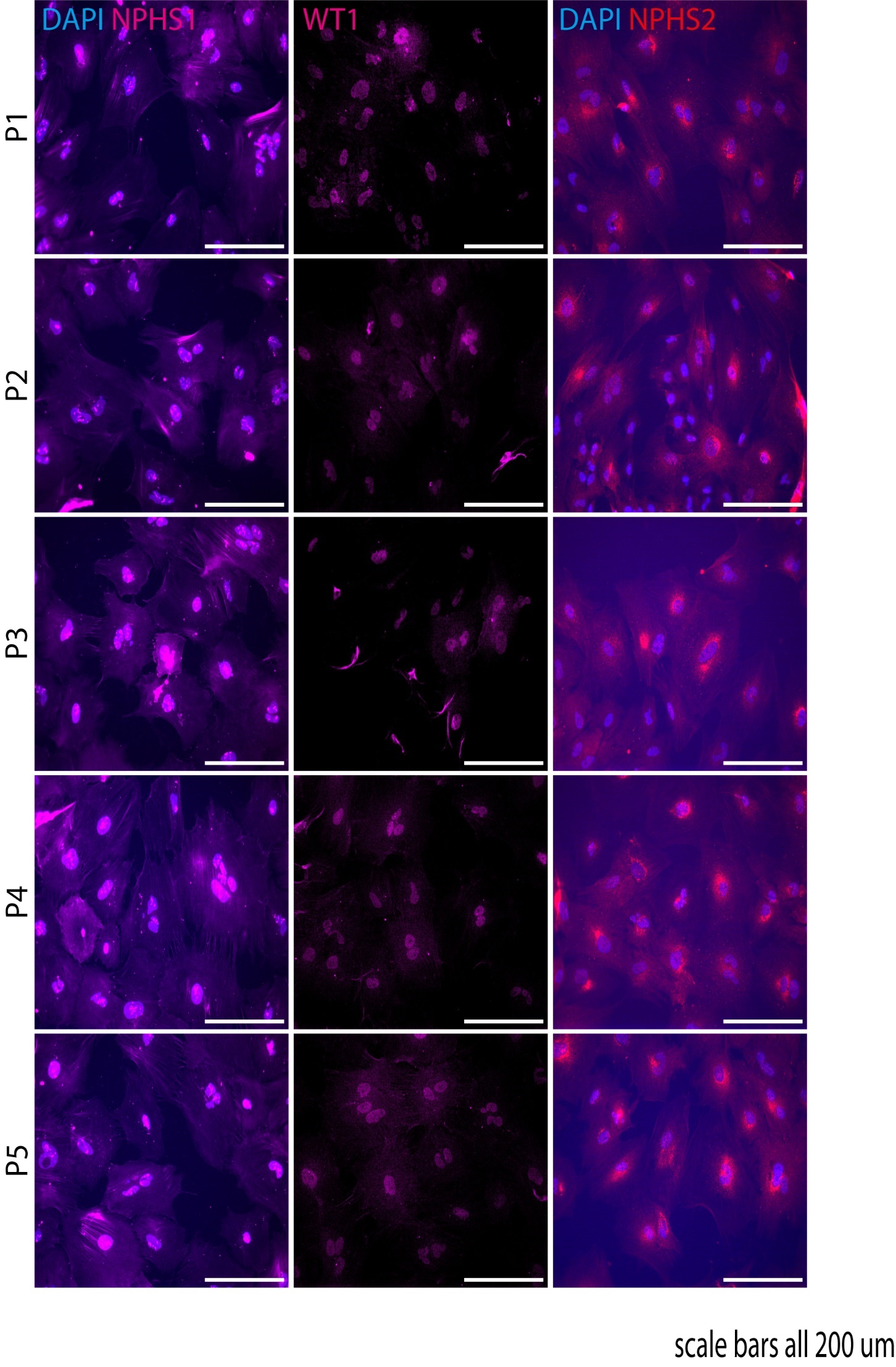


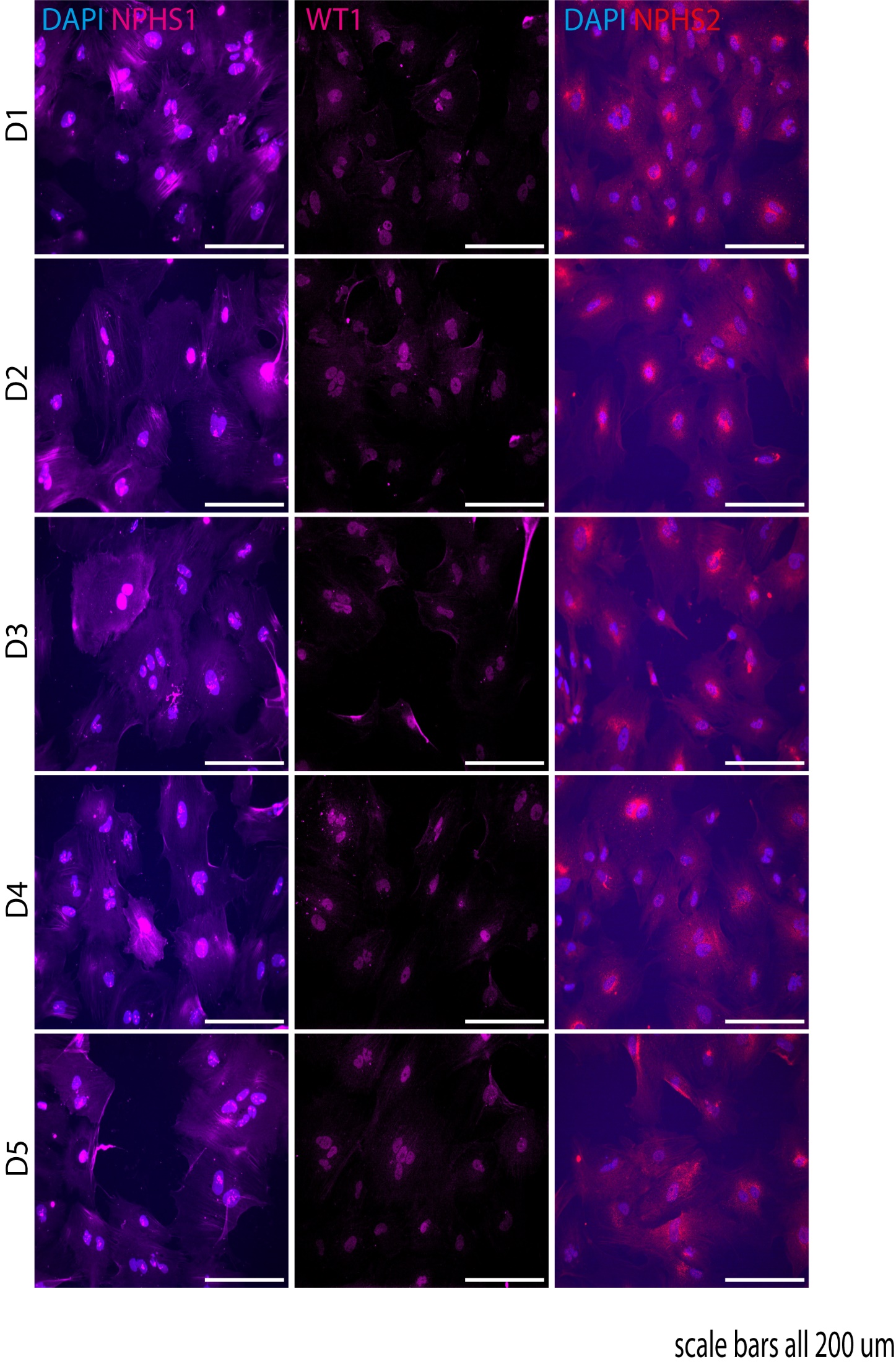


**Supplemental figure 1: Human iPSC-derived podocytes from patients and donors show differential consistency in podocyte-specific markers.** (P1-P5 + D1-D5) Representative images of podocyte specific marker expression in iPSC-derived podocytes from patients (P1-P5) and their respective donors (D1-D5) at D20 of differentiation. iPSC-derived podocyte marker expression included nephrin (NPHS1)(left column), Wilms’ Tumor 1 (WT1)(middle column), and podocin (NPHS2)(right column). IPSC-derived podocytes from patient and donors showed consistent differential status for NPHS1, WT1 and NPHS2 (N=3). Scale bars represent 200 µm. Brightness and contrast were adapted on whole images.


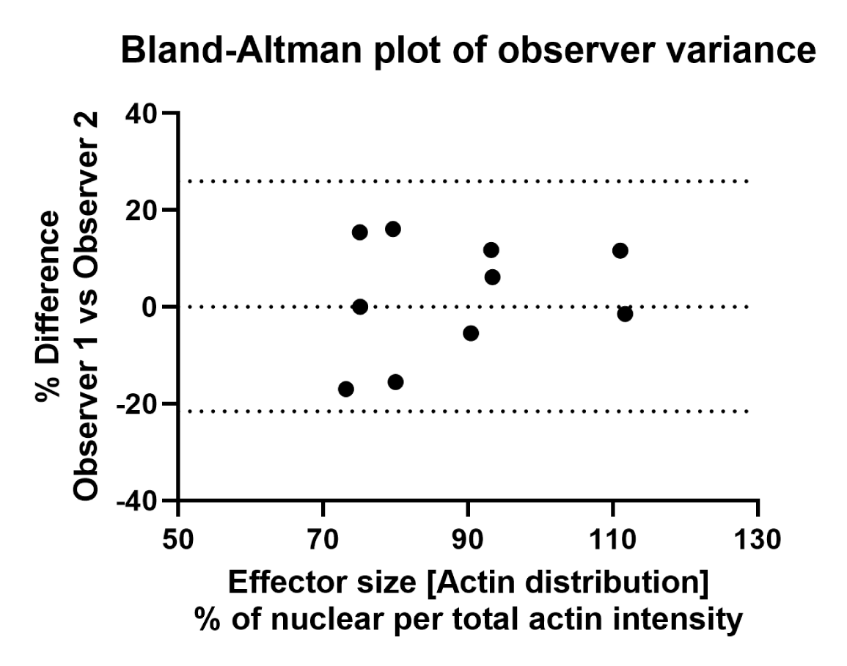


| Bias | Standard deviation | Pearson R^2^ | Spearman rho (*r*) |
| --- | --- | --- | --- |
| 2,18% | 12,13% | 0.051 (p=0.53) | 0.079 (p=0.84) |

**Supplemental figure 2. Observer variance of manual cellular annotation of cytoskeletal distribution (FAR) in human iPSC-derived podocytes.** The Bland-Altman plot shows the percentage of difference between the 2 observers (y-axis) vs the effector size (actin distribution (% of nuclear per total actin intensity))(x-axis). Mean observer variance (bias) was 2,2% with a standard deviation of 12,1%. No significant correlation between variance and effector size was observed.

**
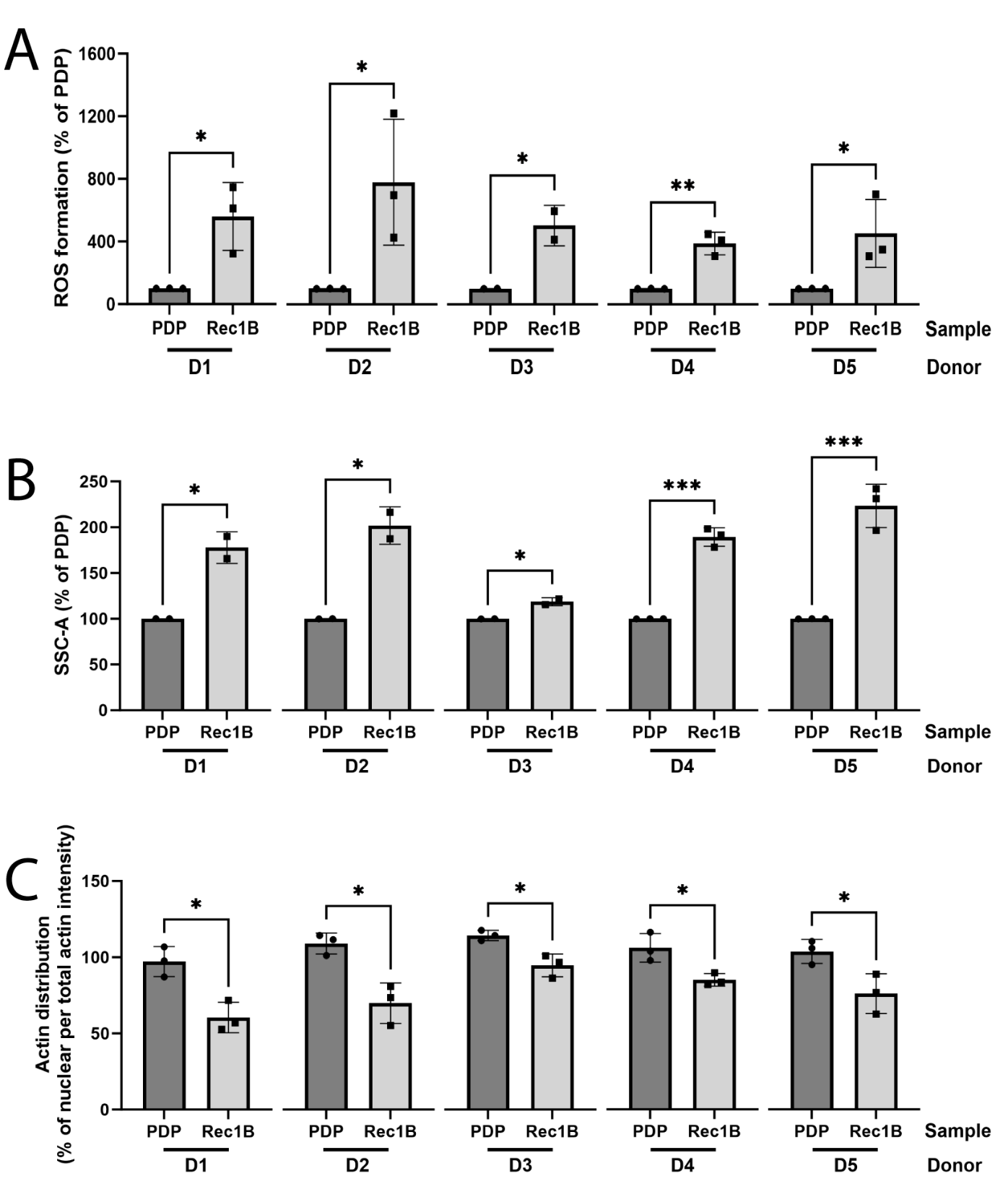
Supplemental figure 3. Evaluation of podocyte injury assays in iPSC-derived podocytes.** (A) ROS formation was measured in donor iPSC-derived podocytes exposed to either healthy PDP or the positive control Rec1B. Statistically significant differences were observed in all donor podocytes (N=2-3). (B) Cellular granule formation (SSC) was measured in donor iPSC-derived podocytes exposed to either healthy PDP or the positive control Rec1B. Statistically significant differences were observed in all donor podocytes (N=2-3). (C) Actin redistribution (FAR) was measured in donor iPSC-derived podocytes exposed to either healthy PDP or the positive control Rec1B. Statistically significant differences were observed in all donor podocytes (N=3). PDP: pooled donor plasma; SSC-A: side scatter area. * P≤0.05; ** P≤0.01; *** P≤0.001 (Student’s t-test).


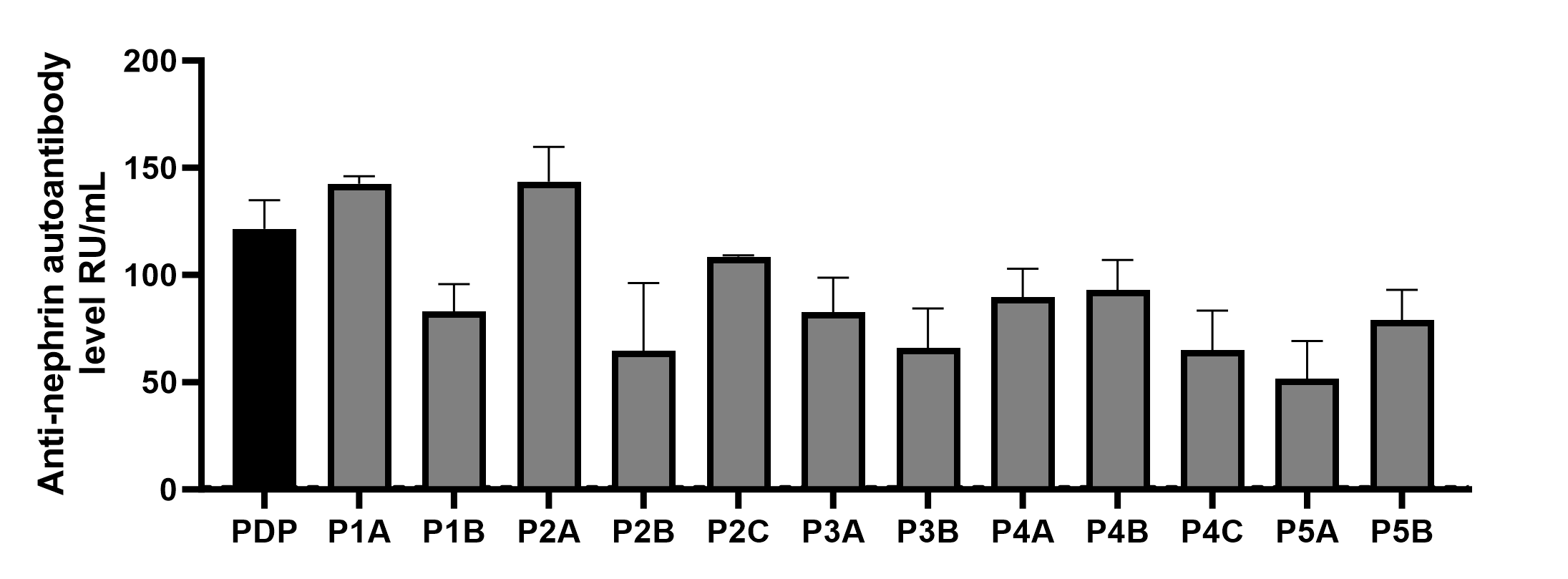


**Supplemental figure 4. Anti-nephrin autoantibody levels in plasma of kidney donors and patients.** Anti-nephrin autoantibody levels were measured by ELISA and set relative (RU/mL). None of the patient samples were found to be statistically significantly higher in both the pre-Tx as well as the post-Tx patient samples (as statistical test, an ANOVA was performed).

**
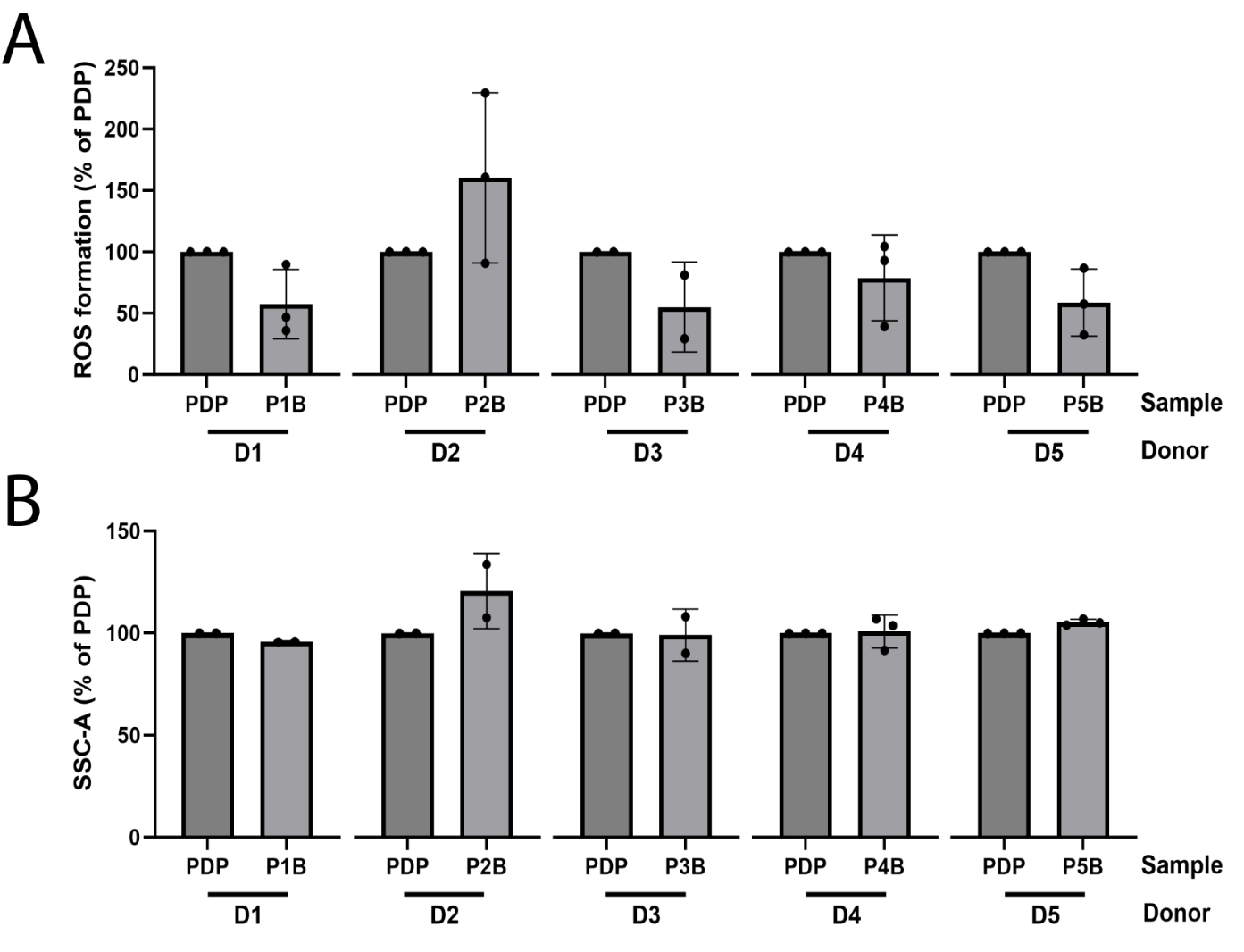
Supplemental figure 5. Detecting rFSGS podocyte injury in iPSC-derived podocytes.** (A) ROS formation was measured in donor iPSC-derived podocytes exposed to either healthy PDP or their respective post-Tx patient plasmas. No statistically significant differences were observed (N=2-3). (B) Cellular granule formation (SSC) was measured in donor iPSC-derived podocytes exposed to either healthy PDP or their respective post-Tx patient plasmas. No statistically significant differences were observed (N=2-3). PDP: pooled donor plasma; SSC-A: side scatter area. As statistical tests, Student’s t-tests were performed.


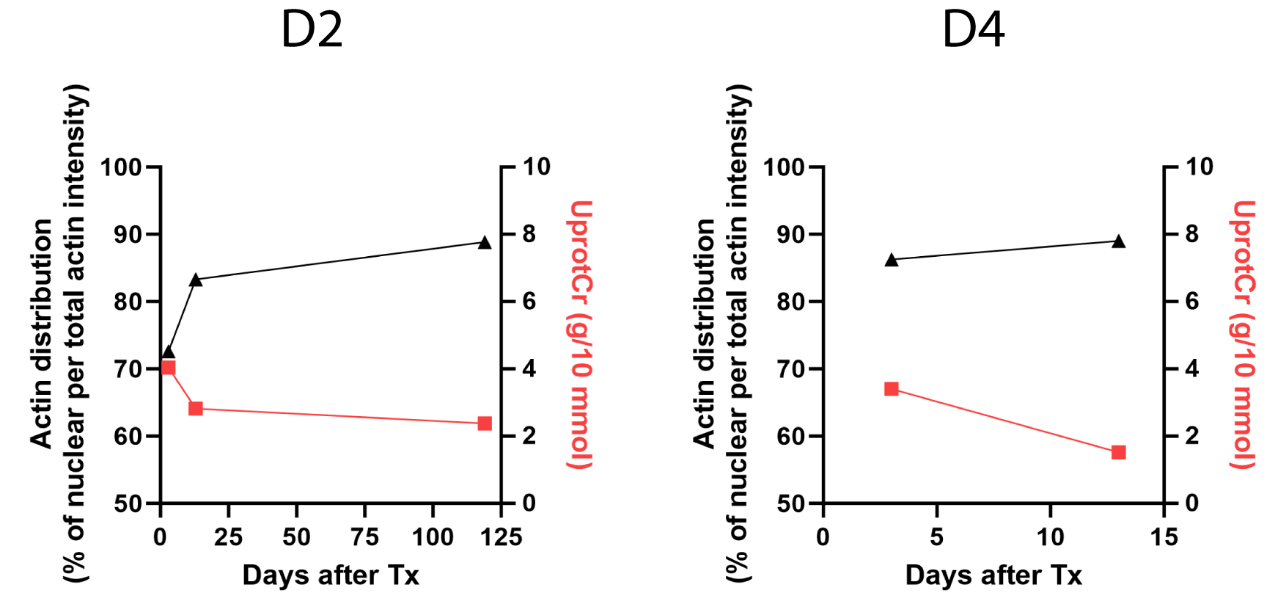


**Supplemental figure 6. Individual disease course modeling using donor iPSC-derived podocytes.** Donor iPSC-derived podocytes of D2 and D4 were exposed to all post-Tx patient plasma of P2 and P4, respectively, to model disease recurrence and subsequent (partial) remission. FAR was plotted over time as well as urinary protein creatinine ratios (UProtCr) to show patient disease status at time of blood sampling. Lower levels of UProtCr coincided with higher levels of actin distribution (so lower levels of podocyte injury), suggesting podocyte injury measured with our FAR assay mimics *in vivo* disease status and recovery.


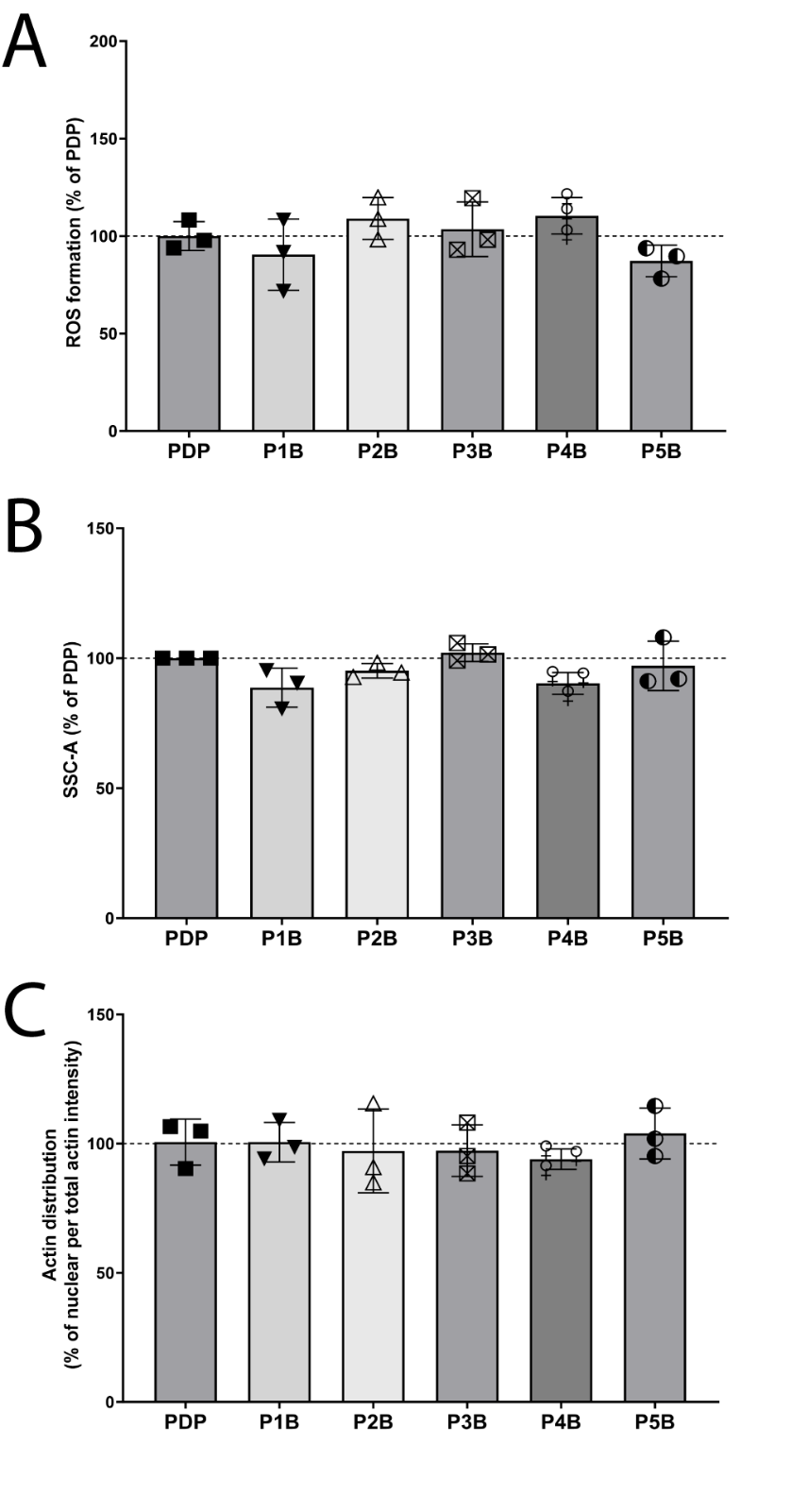


**Supplemental figure 7. FSGS recurrence modelling using conditionally immortalized podocytes (ciPOD).** (A) ROS formation was measured in ciPODs exposed to either healthy PDP or their respective post-Tx patient plasma samples (N=3). No statistically significant difference was observed. (B) Cellular granule formation (SSC) was measured in ciPODs exposed to either healthy PDP or their respective post-Tx patient plasma samples (N=3). No statistically significant difference was observed. (C) Actin rearrangement (FAR) was measured in ciPODs exposed to either healthy PDP or post-Tx patient plasma samples*.* No statistically significant difference was observed (N=3). PDP: pooled donor plasma; SSC-A: side scatter area. As a statistical test, an ANOVA was performed.
