# Supplementary material for: Personalized disease recurrence modeling using iPSC-derived podocytes in patients with idiopathic nephrotic syndrome
Source: Nephrol Dial Transplant. 2025 Feb 28;40(9):1736–45. doi: 10.1093/ndt/gfaf045 (PMC12451694; doi:10.1093/ndt/gfaf045)
Supplement: gfaf045_Supplemental_Files [file gfaf045_Supplemental_Files.zip › 875 NDT_Supplemental materials methods_Personalized disease recurrence modeling using iPSC-derived podocytes in patients with idiopathic nephrotic syndrome_final_cle.docx]

**Supplemental Materials & Methods**

## Cell culture

iPSC generation Recipient and kidney donor iPSCs were generated using PBMCs obtained from blood at the Radboudumc Stem Cell Technology Center through the using the CytoTune™-iPS 2.0 Sendai Reprogramming Kit (Thermo Fisher) as previously described by Jansen et al. (2022, Development). iPSC colonies were picked, expanded and examined on both gene and protein levels for activation of stem cell markers (SOX2, LIN28, NANOG, DNMT3B, SSEA4, Oct3/4, Nanog and Tra-1-81) to confirm pluripotency.

iPSC maintenance culture Human iPSCs were cultured using Essential™ 8 (E8) medium (Gibco, Thermo Fisher) supplemented with E8 supplement (50x, Gibco) and 0.5% (v/v) antibiotic-antimycotic (Gibco) on Geltrex-coated (Thermo Fisher) cell culture plates (Greiner) at 37°C, 5% (v/v) CO_2_. Upon 70-90% confluency, iPSCs were washed three times with PBS and subsequently passaged in colonies using 0.5 mM EDTA (Thermo Fisher) in PBS for 5 min at room temperature (RT). For cell seeding, iPSCs were washed three times with PBS and subsequently disassociated into single cells using TrypLE Select Enzyme (Thermo Fisher) for 3 min at 37°C. When not passaging, the medium was refreshed every two or three days.

Conditionally immortalized podocytes Human conditionally immortalized podocytes (ciPOD) were cultured in DMEM-HAM’s F12 (Gibco) supplemented with 5 µg/ml insulin, 5 µg/ml transferrin and 5 ng/ml selenium (ITS, Sigma), 10% fetal calf serum (FCS, Gibco) and 1% (v/v) penicillin/streptomycin (Gibco) on uncoated culture plates (Greiner) at 33°C to allow for proliferation. For differentiation, cells were seeded at 10,000 cells/cm^2^ on 1% (w/v) Gelatin-coated (Sigma) cell culture plates (Greiner) and cultured at 33°C, 5% (v/v) CO_2_ for 1 day. After 24 hours, cells were transferred to 37°C, 5% (v/v) CO_2_ to halt proliferation and start differentiation into the podocyte(-like) fate for another 7 days. The ciPODs used in this manuscript were kindly provided by Prof. dr. Saleem (University of Bristol, UK) (Saleem *et al.* 2002 JASN).

## IPSC differentiation protocol

A hybrid directed differentiation protocol based on Rauch et al. (2018 Plos One) and Musah et al. (2018 Nat Protoc) was developed to differentiate iPSCs into podocytes. iPSCs were seeded at 15,000 cells/cm^2^ on Geltrex-coated cell culture plates in E8 medium supplemented with E8 supplement, 0.5% (v/v) antibiotic-antimycotic and 1x RevitaCell (Gibco), henceforth known as D-1. The next day, D0, differentiation of iPSCs into intermediate mesoderm (IM) was initiated by the addition of Essential™ 6 (E6) medium (Gibco), supplemented with 15 ng/ml human recombinant bone morphogenetic protein 7 (R&D Systems), 10 ng/ml human recombinant activin A (R&D Systems) and 100 nM retinoic acid (Sigma-Aldrich). The medium was refreshed every two or three days. After 7 days, at D7, cells were washed three times with PBS and subsequently disassociated into single cells using TrypLE Select Enzyme (Thermo Fisher) for 5 min at 37°C. Cells were re-seeded at 20,000 cells/cm^2^ on 1% (w/v) Gelatin-coated (Sigma) cell culture plates (Greiner) in podocyte medium (DMEM-HAM’s F12 (Gibco) supplemented with 5 µg/ml insulin, 5 µg/ml transferrin and 5 ng/ml selenium (ITS, Sigma), 10% fetal calf serum (FCS, Gibco)1% (v/v) penicillin/streptomycin (Gibco)) containing the aforementioned growth factors human recombinant bone morphogenetic protein 7 (15 ng/ml, R&D Systems), human recombinant activin A (10 ng/ml, R&D Systems) and retinoic acid (100 nM, Sigma-Aldrich). Re-seeding halts cell proliferation during IM development and initiates maturation into the podocyte fate. From D10, cells were cultured in podocyte medium supplemented with 1% (v/v) non-essential amino acids (NEAA, Gibco) without the addition of growth factors. At day D20, iPSC-derived podocytes were considered mature and used for experiments.

## Experimental treatment

iPSC-derived podocytes or human ciPODs were treated with either 10% (v/v) plasma, serum or plasmapheresis material in serum-free podocyte medium for 24 hours after which subsequent end-point measurements were performed. Serum-free podocyte medium consisted of DMEM-HAM’s F12 (Gibco) supplemented with 5 µg/ml insulin, 5 µg/ml transferrin and 5 ng/ml selenium (ITS, Sigma) and 1% (v/v) penicillin/streptomycin (Gibco)), anti-coagulants heparin (100 µg/ml, Sigma) and PPACK (10 µM, Santa Cruz Biotechnology).

## ROS formation

iPSC-derived podocytes were washed with HBSS once and loaded with 10 μM CM-H2DCFDA (ROS probe, Invitrogen) dissolved in podocyte medium for 30 minutes. After loading, cells were washed with HBSS twice and exposed to experimental treatments. After 24 hrs, ROS formation was measured by fluorometry (485 excitation/535 emission) with a Victor 3 V Multilabel Plate reader (PerkinElmer). As a positive ROS control, 500 μM hydrogen peroxide was used in all experiments. Background levels for background correction were determined by measuring CM-H2DCFDA signal in unloaded cells exposed to (control) podocyte medium.

## Granule formation analysis using flow cytometry

IPSC-derived podocytes were washed with HBSS once and disassociated using TrypLE Select Enzyme for 5 min at 37°C. After dissociation, cells were washed in HBSS and resuspended in 1% (v/v) Bovine Serum Albumin (BSA, Sigma) in PBS. Cells were measured with a flow cytometer (Novocyte 3000 flow cytometer, Agilent), as previously described by den Braanker et al. (2021 Nephrol Dial Transplant). In short, granule formation in iPSC-derived podocytes was quantified using sideward scatter.

## Fiji quantification

Fluorescence images obtained from plasma/serum/plasmapheresis exposure experiments were analyzed using custom-made macros in the free open-software Fiji/ImageJ (Fiji version 1.53t) (Zenodo link: 10.5281/zenodo.12517187). First, the cellular area was manually outlined using the phalloidin staining as a selection for total cellular actin area and intensity measurements. Second, using a readily available Fiji threshold algorithm, actin specific area and intensity were determined. Lastly, again using a readily available Fiji threshold algorithm, nuclear actin specific area and intensity were determined using the DAPI signal as area selection. The same area selection algorithms were used for both the actin specific as well as nuclear actin area and intensity measurements. Nuclear actin intensity per total cellular actin intensity was used as a way to measure actin distribution rearrangements (FAR). Actin cytoskeleton quantification (FAR) was performed in five podocytes that were manually outlined per cell culture well (technical repeat). Technical repeats (NT) were performed in triplicate. 15 podocytes were thus analyzed in 3 different wells per experiment. Biological repeats (NB) are classified as one experiment analyzed per cell culture plate and were also performed in triplicate. Biological repeats always had different passages of maintenance iPSCs and subsequent iPSC-derived podocytes differentiations and were therefore performed on different dates.

The total amount of podocytes analyzed per N=3 is therefore 5 podocytes x N^T^ x N^B^ (5 x 3 x 3 = 45). As an internal quality control for automatic threshold selection, images containing the respective ROI selection were automatically created for post-processing analysis to ensure correct ROI selection in all conditions.

## Immunofluorescent staining

iPSC-derived podocytes cultured in 96-well plates (Greiner) were washed with HBSS (Gibco) and fixed in 2% (w/v) PFA for 10 min at RT. After washing twice with HBSS, cells were incubated with blocking permeability buffer (BPB) (10% (v/v) donkey serum, 0.06% Triton X-100 (Sigma) in PBS) for 2 hrs at RT. After blocking and permeabilization, cells were incubated with primary antibodies in BPB (1:100) overnight at 4°C. The next day, cells were washed twice with PBTX (0,3% Triton X-100 in PBS) and exposed to secondary antibodies in PBTX (1:200) for 2 hours at RT. Plates were covered in aluminium foil to prevent fluorescent fading. Next, cells were washed twice with HBSS and mounted using Fluormount-G®. Finally, images were captured using a Leica DMI6000B high-content microscope. Primary and secondary antibodies and their respective working dilutions can be found in supplementary materials table 2 (S2).

## Anti-human nephrin ELISA

Anti-nephrin autoantibody level were measured and quantitated using ELISA. In short, Nunc MaxiSorp ELISA plates (Thermo Fisher) were coated with 100 ng per well using recombinant human nephrin protein (extracellular domain, #17757-H08H, Sino Biological), diluted in 100 µl of Biolegend coating buffer and incubated overnight at 4°C. After washing with TBS-T, plates were blocked with SuperBlock™ blocking buffer (Thermo Fisher) and then incubated for 2 hours at room temperature (RT) with 100 μl patient’s samples diluted at 1:100 in blocking buffer with 0.1% Tween. After washing, 100 μL of goat HRP-conjugated anti-human IgG (1:10.000, Southern Biotech) in blocking buffer with 0.1% Tween was added for 2 hours at RT. Wells were again washed, then TMB substrate solution was added, followed by stop solution 10 min later and the absorbance at 450 nm was measured. A standard curve for relative IgG titer was made using a highly nephrin-positive serum whose titer was arbitrary defined as 1000 units (RU/mL).

**Supplemental Table S1.** Reagent and resource table

| **Chemicals, Peptides, ELISAs and Recombinant Proteins** | | |
| --- | --- | --- |
| Essential 8 FLEX Medium | Thermo Fisher | Cat#A2858501 |
| Essential 6 Medium | Thermo Fisher | Cat#A1516401 |
| UltraPure EDTA (0.5M) pH 8.0 | Thermo Fisher | Cat#15575020 |
| Geltrex LDEV-free, hESC-Qualified, reduced growth factor basement membrane matrix | Thermo Fisher | Cat#A1413302 |
| Antibiotic-Antimycotic | Thermo Fisher | Cat#15240062 |
| Gelatin from porcine skin | Sigma Aldrich | Cat#G2500-100G |
| Heparin sodium salt from porcine intestinal mucosa | Sigma Aldrich | Cat#H4784 |
| PPACK dihydrochloride | Santa Cruz Biotechnology | Cat#sc-201291 |
| Recombinant Human BMP-7 Protein | R&D Systems | Cat#354-BP-010 |
| Human Activin A | Milteyni Biotec | Cat#130-115-010 |
| Retinoic acid | Sigma Aldrich | Cat#2625-100MG |
| MEM Non-Essential Amino Acids Solution | Thermo Fisher | Cat#11140050 |
| TrypLE Select Enzyme, no phenol red | Thermo Fisher | Cat#12563-029 |
| RevitaCell Supplement | Thermo Fisher | Cat#A2644501 |
| DMEM/F-12 | Thermo Fisher | Cat#11320074 |
| Human EGF | Sigma Aldrich | Cat#E9644 |
| Insulin-transferrin-sodium selenite media supplement | Sigma Aldrich | Cat#I1884-1VL |
| Penicillin-Streptomycin (5000 U/mL) | Thermo Fisher | Cat#15070063 |
| Fetal Bovine Serum, heat inactivated | Thermo Fisher | Cat#16140071 |
| Nunc™ MaxiSorp™ ELISA Plates, Uncoated | BioLegend | Cat#423501 |
| Recombinant Human Nephrin Protein | SinoBiological | Cat#17757-H08H |
| ELISA Coating Buffer (5X) | BioLegend | Cat#421701 |
| SuperBlock™ Blocking Buffer | Thermo Fisher | Cat#37515 |
| Goat Anti-Human IgG-HRP | Southern BioTech | Cat#2040-05 |
| **Critical Commercial Assays** | | |
| PSC Cryopreservation Kit | Thermo Fisher | Cat#351-FS-050 |
| **Deposited Data** | | |
| Scripts and codes for data analysis | This paper; deposited on Zenodo | (10.5281/zenodo.12517187). |
| **Experimental Models: Cell Lines** | | |
| Human induced pluripotent stem cell line P1 | SCTC Radboud UMC, The Netherlands | iPS 19_103 |
| Human induced pluripotent stem cell line P2 | SCTC Radboud UMC, The Netherlands | iPS 20_013 |
| Human induced pluripotent stem cell line P3 | SCTC Radboud UMC, The Netherlands | iPS 20_014 |
| Human induced pluripotent stem cell line P4 | SCTC Radboud UMC, The Netherlands | iPS 19_112 |
| Human induced pluripotent stem cell line P5 | SCTC Radboud UMC, The Netherlands | iPS 20_040 |
| Human induced pluripotent stem cell line D1 | SCTC Radboud UMC, The Netherlands | iPS 19_102 |
| Human induced pluripotent stem cell line D2 | SCTC Radboud UMC, The Netherlands | iPS 20_021 |
| Human induced pluripotent stem cell line D3 | SCTC Radboud UMC, The Netherlands | iPS 20_015 |
| Human induced pluripotent stem cell line D4 | SCTC Radboud UMC, The Netherlands | iPS 19_111 |
| Human induced pluripotent stem cell line D5 | SCTC Radboud UMC, The Netherlands | iPS 20_41 |
| Conditionally immortalized human podocyte cell line | Prof. Saleem, Bristol, UK | ciPOD |
| **Software and Algorithms** | | |
| ImageJ version Fiji 1.51n | National Institutes of Health, USA | <https://imagej.nih.gov/ij/> |
| Adobe Illustrator CC 2021 | Adobe Systems Inc. | RRID:SCR_010279 |
| Adobe Photoshop CC 2021 | Adobe Systems Inc. | RRID:SCR_014199 |
| GraphPad Prism version 9.5 | GraphPad Software Inc. | RRID:SCR_002798 |

**Supplemental Table S2.** Antibodies used for immunofluorescence staining.

| **Primary antibody** | **Working dilution** | **Secondary antibody** | **Working dilution** |
| --- | --- | --- | --- |
| Human Nephrin Antibody (AF4269, R&D Systems) | 1:100 | Donkey anti-sheep Alexa Fluor™ 647 (A21448, Thermo Fisher) | 1:200 |
| Anti-Podocin antibody (P0372, Sigma Aldrich) | 1:100 | Donkey anti-rabbit Alexa Fluor™ 568 (A10042, Thermo Fisher) | 1:200 |
| Recombinant Anti-Wilms Tumor Protein antibody (ab89901, Abcam) | 1:100 | Donkey anti-mouse Alexa Fluor™ 647 (A31571, Thermo Fisher) | 1:200 |
| Anti-Synaptopodin antibody (65194, Progen) | 1:100 | Donkey anti-mouse Alexa Fluor™ 488 (A21202, Thermo Fisher) | 1:200 |
| Monoclonal Anti-Vinculin antibody (V9131, Sigma) | 1:100 | Donkey anti-mouse Alexa Fluor™ 647 (A31571, Thermo Fisher) | 1:200 |
| Anti-CD2AP antibody (HPA003326, Sigma) | 1:100 | Donkey anti-rabbit Alexa Fluor™ 647 (A31573, Thermo Fisher) | 1:200 |
| Vimentin monoclonal antibody (MA5-11883, Invitrogen) | 1:100 | Donkey anti-mouse Alexa Fluor™ 647 (A31571, Thermo Fisher) | 1:200 |
| Goat Anti-Type IV Collagen-UNLB (1340-01, Southern Biotech) | 1:100 | Donkey anti-Goat Alexa Fluor™ 488 (A-11055, Thermo Fisher) | 1:200 |
| Flash Phalloidin^™^ Green 488 (424201, Biolegend) | 1:100 | n.a. |  |
